# Therapeutic Potential of Natural Pharmacological Agents in the Treatment of Human Diseases

**DOI:** 10.1155/2014/573452

**Published:** 2014-12-28

**Authors:** Kota V. Ramana, Sharad S. Singhal, Aramati B. Reddy

**Affiliations:** ^1^Department of Biochemistry and Molecular Biology, University of Texas Medical Branch, Galveston, TX 77555, USA; ^2^Department of Diabetes and Metabolic Diseases Research, Beckman Research Institute, City of Hope National Medical Center, Duarte, CA 91010, USA; ^3^Department of Animal Sciences, University of Hyderabad, Hyderabad 500046, India

The importance of natural products produced by living organisms from the nature for health and disease treatment has been immense throughout the human evolution. Several natural products derived from plants for thousands of years have been traditionally used to treat various types of human illnesses including general injuries, wound healing, and pain. Natural products have been shown to possess significant pharmacological activities that regulate various vital cell signaling pathways that cause mitogenic, cytotoxic, and genotoxic reactions leading to various disease pathologies. Modern synthetic and combinatorial chemistry associated with the new technological tools such as genomics, proteomics, and metabolomics paved wider use of natural products. Nowadays, most of the natural products are processed and developed as potential pharmacological agents with effective antioxidative, antimitotic, anti-infective, anti-inflammatory, antiangiogenic, and anticarcinogenic properties. In fact, some natural products have been employed as lead compounds to obtain highly biologically relevant semisynthetic pharmacological derivatives with increased efficiency and efficacy for the therapeutic use. The prosperity of knowledge we are assembling from the past several decades will significantly inspire us to develop these natural products as novel potential drugs for future therapeutic strategies.

This special issue compiles 38 excellent manuscripts, including clinical studies, research articles, and reviews, which provides comprehensive evidence demonstrating the significance of natural plant and microbial-derived products in human health and disease.

The review articles of this special issue discuss the importance of some natural products derived from various types of plant species that are involved in maintaining the human health and their potential role in preventing various pathological conditions ranging from bacterial infections to cancer. An excellent review article by M. Gangwar et al. described in depth pharmacological use of* Mallotus philippinensis* Muell. Arg (Euphorbiaceae), a widely grown perennial shrub in the outer Himalayan region. This review describes how various natural products such as flavonoids, phenols, coumarins, and terpenoids derived from these plant species exhibit various medicinal properties and how these products could be used in the modern medicine. A comprehensive review article by T. Sham et al. discussed the significance and popularity of traditional Chinese medicine in controlling hyperlipidemia. In particular, this article describes underlying mechanisms of nine specific single Chinese herbs and seven classical formulae of traditional Chinese medicine based on recent experimental and clinical data on hyperlipidemia. Another interesting review article by M. H. Boskabady et al. discussed the significant pharmacological effects of* Carum copticum L*. plant (the celery; also known as “Ajwain” in India). In particular, this review describes how these plant products have been extensively used in natural medicine for thousands of years to treat respiratory and digestive problems. N. Gautam et al. in their review article described the mechanisms of action of different essential oils and their constituents in the treatment of various cancers. In particular, the authors have nicely highlighted how multiple pathways of cell growth and death are regulated by the essential oils and their constituents* via* modulating the antioxidant enzymes, oxidative stress, redox sensitive transcription factors, and protein kinases. A brief review article by J. Li et al. describes anti-inflammatory, antiviral, and antiallergic actions of glycyrrhizic acid, a triterpene glycoside found in the roots of licorice (*Glycyrrhiza glabra*) plants. Glycyrrhizic acid has been shown to exhibits anti-inflammatory effects by inhibiting NF-kB mediated production of inflammatory cytokines and chemokines. In particular, this review discusses the pharmacological actions of glycyrrhizic acid in hepatic diseases and its mechanisms of actions in cellular and animal studies. Another review by S. Sreekumar et al. discussed the pharmacological properties of pomegranate fruit. In particular, they have discussed a protective role of methanol extract of pericarp of pomegranate on cardiac and skeletal tissues and as an anticancer agent(s) against various cancer types. These review articles thus provided widespread information on the use of natural plant products in various human diseases.

The research article by M. H. Boskabady and L. G. Mhtaj investigates the effects of* Zataria multiflora* on animal models of chronic obstructive pulmonary disease (COPD). In this study, hydroethanolic extract of* Z. multiflora* was examined for its anti-inflammatory role in preventing COPD in guinea pigs. These studies indicate that* Z. multiflora* could prevent symptoms of COPD and its effects are comparable to the effects of dexamethasone at the concentrations tested. Studies by F. L. Westphal et al. reported the induction of pleurodesis, a method that causes the membranes around the lungs to stick together and prevent the fluid buildup, by copaiba oil in rats. In this study, the effect of induction of pleurodesis by copaiba oil was compared with the silver nitrate. Their results indicate that both copaiba oil and silver nitrate promoted pleurodesis in rats. However, overall better results were seen in the copaiba group as compared to the silver nitrate group. Silver nitrate group demonstrated greater aggression to the pulmonary parenchyma as compared to copaiba oil. In another research article, A. Bharathi et al. examined* in silico* molecular docking and* in vitro* antidiabetic activity of novel 10-chloro-4-(2-chlorophenyl)-12-phenyl-5,6-dihydropyrimido[4,5-a]acridin-2-amines (3a–3f). These studies indicate that compound 3e shows selective inhibition of *α*-amylase and *α*-glucosidase.

A research article by S. Pal et al. examined the protective effect of ethanol extract of* Alocasia indica* tuber on hepatotoxicity in alcohol treated rat model. These studies report that* A. indica* tuber extract prevents alcohol-induced liver biomarkers such as serum alanine aminotransferase (ALT), aspartate aminotransferase (AST), *γ*-glutamyl transpeptidase (*γ*-GT), and total bilirubin levels. Further, this plant extract also significantly restored the alcohol-induced levels of malondialdehyde, nitric oxide, glutathione, catalase, and superoxide dismutase suggesting that supplementation of* A. indica* tuber extract could be protective towards alcohol-induced liver injury. In another research article, A. Sudha and P. Srinivasan identified the DPPH (2,2-diphenyl-1-picrylhydrazyl) free-radical scavenging constituents from methanol extract of L. nodiflora using bioassay-guided fractionation. They found that bioactive compound 2-(3,4-dimethoxyphenyl)-5-hydroxy-7-methoxy-4H-chromen-4-one (5-hydroxy-3′,4′,7-trimethoxyflavone) isolated from ethyl acetate fraction of plant leaves extract possesses strong antioxidant activity. Similarly, M. Khanapur et al. also reported the antioxidant and antimitogenic activities of flower extracts of* Nyctanthes arbortristis*.

A research article by M. F. Mahomoodally and D. P. Sreekeesoon reported interesting data on the use of natural therapies for the pediatric health care in Mauritius. Based on the interview of parents, they tabulated use of various natural products in pediatric patients of different illnesses. This is one of the first studies to gather primary folk knowledge on the use of plant- and animal-based therapies as natural pharmacological agents for child care. Anticancer properties of the leaves of the medicinal plant* Sesbania grandiflora* were reported by S. Pajaniradje et al. in their research article. Out of five different solvent fractions from the leaves of* S. grandiflora* tested for anticancer activities, methanolic fraction was found to be potent growth suppresser of the human A549 non-small cell lung cancer cell line. They found that methanolic fraction of* S. grandiflora* activates caspase-3 and decreases mitochondrial membrane potential, cyclin D1, and NF-kB in A549 cells. These studies thus indicate that* S. grandiflora* plant products could be used to prevent lung cancer. Research article by M. P. De Luca et al. demonstrated the antimicrobial properties of propolis varnish against cariogenic bacteria. The paper reports that propolis varnish formulations have satisfactory antimicrobial activity against cariogenic bacteria with low cytotoxicity.

An interesting research study by L. Mei et al. reported antidepressive actions of Pycnogenol (PYC), a natural plant extract from the bark of* Pinus pinaster Aiton*. These studies indicate that oral administration of PYC prevents depression-like behavior in a chronic corticosterone-treated mice model of depression. Another research study by P. H. M. Nunes et al. reported antioxidative and pharmacological actions of various bark extracts of plant* Terminalia fagifolia* Mart. & Zucc using rat models of ethanol-induced ulcers and toxicity. In another study, M. A. Hossain et al. investigated the antimicrobial activity of* Nymphaea tetragona* alone or in combination with antibiotics against different strains of* Salmonella* bacteria. Their results indicate that combination of ethyl acetate fraction of* N. tetragona* extract along with antibiotics could be useful to combat drug-resistance* Salmonella* infections. Z. Li et al., in their research article, demonstrate antitumor activity of icaritin (ICT), a hydrolytic product of icariin from* Epimedium* genus. By using Burkitt lymphoma cell lines such as Raji and P3HR-1 they found that icaritin prevents cell proliferation and induces apoptosis* via* activation of caspase-8, caspase-9 and cleavage of PARP and shifting the ratio of Bcl-2/Bax. Further, these studies indicate that icaritin induces cell cycle arrest by increasing the percentage of cells in S-phase and reducing the population of cells in G0/G1 phase. Thus, these studies provide preliminary evidence for the use of icaritin in lymphoma treatment.

Another research article by R. Sudan et al. demonstrated antioxidant and immunomodulatory actions of* Arisaema jacquemontii* plant extracts in mice. In particular, these studies indicate that* A. jacquemontii* leaves have considerable antioxidant and immunomodulating potential as compared to tuber and fruits of the plant. Similarly, another research study by D. Iqbal et al. reported antioxidant, genoprotective, antilipoperoxidative, and HMG-CoA reductase inhibitory properties of traditional medicinal plant,* Ficus palmata Forsk*. In particular, they report that both* F. palmata* bark aqueous extract and* F. palmata* leaves methanolic extracts exhibit significant radical scavenging and antioxidant capacity. Further, they report that* F. palmata* bark aqueous extract inhibits lipid peroxidation and HMG-CoA reductase activity. In another study, D. Natarajan et al. reported the antimicrobial potential of solvent leaf extracts of* P. wightianus*. By using 11 human bacterial pathogens and 4 fungal pathogens they showed that leaf extracts of* P. wightianus* possess antimicrobial activity.

A. Mushtaq et al. examined ethanol, methanol, and aqueous extracts of* Eremurus himalaicus* for their hypoglycaemic effects in normal Wister Albino rats. They report that as compared to alcohol extracts aqueous extract exhibits significant hypoglycaemic activity in normoglycaemic rats. Similarly, P. Kumar et al. reported that aqueous extract of* Trigonella foenum*-graecum seeds (AqE-TFG) prevents fat accumulation and dyslipidemia in high fat diet-induced obese rats. Based on the results from this study, they propose that reduction of impaired lipid digestion and absorption, improved glucose and lipid metabolism, enhanced insulin sensitivity, increased antioxidant defense, and decreased lipogenic enzymes could be responsible for preventive effects of AqE-TFG on fat accumulation and dyslipidemia. In the same lines, N. Anupama et al. in their research study examined the anti-inflammatory effects of dried fruit methanol extract of plant* Carissa carandas* on carrageenan-induced hind paw edema in rats. Another report by A. Bharathi et al. reported* in vitro* larvicidal and antioxidant activities of dihydrophenanthroline-3-carbonitrile derivatives. A decent research study by N. D. Mahmood et al. investigates hepatoprotective activity of methanol extract of* M. calabura* leaves (MEMC) in rat models of liver injury. Their results indicate that as compared to N-acetylcysteine MEMC exerts potential hepatoprotective activity as determined by ALT and AST enzymes levels and histological and radical scavenging assays in paracetamol-induced hepatotoxic rats.

In continuation with several reports on antimicrobial effects of natural products in this issue, G. Simonetti et al. also reported their research findings that grape seed extracts (GSEs) obtained from wine and table cultivars of* Vitis vinifera* L. possess anti-Candida activities. In particular, they indicate that there is a significant correlation between the contents of the flavan-3-ols in GSEs extracts, with a polymerization degree ≥4, and anti-Candida activity. Their conclusion is supported by the studies using an experimental murine model of vaginal candidiasis. In another study, A. Upadhay et al. reported the wound healing property of medicinal plant* Cleome. viscosa* methanol extract (CvME) and its related mechanisms using a Wistar rat cutaneous excision wound model. In this study, the effect of CvME on wound contraction rate, hydroxyproline quantification, and wound granulation were examined. The results indicate that CvME significantly increased wound contraction rate, hydroxyproline content and improved wound granulation. Their data also indicates that* C. viscosa* promotes a wound repair process by inhibiting Smad-mediated collagen production in wound granulation tissues. In another interesting study, O. Carvajal-Zarrabal et al. reported the effect of avocado oil and olive oil supplementation on the hepatic function in sucrose-fed rats. Their results indicate that avocado oil extracted by centrifugation or using solvent exhibited similar hepatic function effects as olive oil and this suggests that consumption of avocado oil is equally beneficial as olive oil for the liver health.

Curcumin has been well established as an antioxidative, anticarcinogenic, and anti-inflammatory agent. Y. H. Siddique et al. reported the effect of curcumin on oxidative stress and apoptosis in the brains of transgenic Drosophila model of Parkinson's disease. The exposure of flies expressing human alpha synuclein to curcumin showed a significant dose dependent delay in the loss of activity pattern, reduction in the oxidative stress, apoptosis, and increase in the life span. Thus, these studies suggest potential use of natural plant product curcumin for the treatment of Parkinson's disease. Similarly, anticervical cancer and antiangiogenic properties of curcumin were also reported by P. Yoysungnoen-Chintana et al. in their research article. Their studies using* nu/nu* nude mice xenografts indicate that high dose of curcumin prevents cervical cancer growth and angiogenesis* via* downregulating VEGF, Cox-2, and EGFR. In an open-label uncontrolled phase-1 pilot study, U. Klickovic et al. examined inducibility of heme oxygenase-1 (HO-1) by orally administered curcumin in healthy male subjects. In addition, these studies also identified a correlation of GT length polymorphism with HO-1. These studies suggest that oral curcumin supplementation has low bioavailability and does not induce HO-1 in human peripheral blood mononuclear cells.

Q. Xu et al. in their article investigated the anticarcinogenic mechanisms of *α*-mangostin, a natural product isolated from the pericarp of the mangosteen fruit. In particular, they report that *α*-mangostin prevents pancreatic cancer cells growth in culture and BxPC-3 xenografts in mice* via* enhancing the apoptosis, modulating the epithelial-mesenchymal transition, and inhibiting the activation of PI3K/AKT signaling pathways. They have also shown that *α*-mangostin decreases the expression of MMP-2, MMP-9, N-cadherin, and vimentin and increased the expression of E-cadherin in pancreatic cancer cells. Thus, these studies indicate that *α*-mangostin could be used as a novel adjuvant therapy or complementary alternative medicine for the treatment of pancreatic cancer. Another research study by X. Xu et al. reported that as compared to naringenin alone the combination of naringenin and *β*-cyclodextrin is more protective in reducing choroidal neovascularization (CNV) in a laser-induced rat model of CNV. The results indicate that treatment of naringenin/*β*-CD complex demonstrated increased water solubility and improved biological activity that leads to inhibition of CNV formation in rats. Their studies also indicate that naringenin/b-cyclodextrin complex significantly prevents the expression of inflammatory markers such as VEGF, COX-2, PI3K, p38MAPK, MMP-2, and MMP-9 in retina and choroid tissues and this suggests that the beneficial effects of the combination towards CNV could be linked to the anti-inflammatory activities of naringenin.

Another research paper by H. Zhang et al. investigated the molecular mechanisms by which phytoestrogen, zearalanol could prevent plasma homocysteine levels in a diet-induced hyperhomocysteinemia rat model. They found that zearalanol elevates cystathionine-synthase, an enzyme responsible for homocysteine metabolism, and reduces nitrative stress. In another research study, C. Xun et al. reported the efficacy of salvianolic acid B on motor function recovery in rats with spinal card injury. These studies indicate that by preventing the activation of IkB-*α* and NF-kB salvianolic acid B could recover motor function after spinal cord injury. The final research article by H. M. Barreto et al. reported antimicrobial activity of various extracts of* Lippia origanoides* against drug resistant* Staphylococcus aureus* strain. Their results suggest that* Lippia origanoides* could be a source of secondary metabolites for use in association with neomycin and amikacin during antimicrobial treatment.

It is obvious from the ancient medical practices, past literature, and current special issue papers that natural products play a major role in human health and prevention or treatment of human diseases. Although, over the past several years, substantial research has shown that natural products have a crucial physiological role in maintaining the human health, the exact nature of their significance and mechanisms by which these natural products regulate cellular homeostasis that maintain cell survival, differentiation, and death leading to pathological consequences required further detailed investigations. Newly developed technologies such as lipid finger printing/lipidomics, metabolomics, and microRNA arrays are important tools that will help to define how the natural plant products provide protection against various pathological conditions. Therefore, the identification and development of important natural products as novel pharmacological agents could help to combat or neutralize the deleterious effects observed during various disease conditions and could be the next important targets for future drug discovery studies.

